# Inactivation of *Staphylococcus aureus* and *Enterococcus faecalis* by a direct-current, cold atmospheric-pressure air plasma microjet^☆^

**DOI:** 10.1016/S1674-8301(10)60037-1

**Published:** 2010-07

**Authors:** Ye Tian, Peng Sun, Haiyan Wu, Na Bai, Ruixue Wang, Weidong Zhu, Jue Zhang, Fuxiang Liu

**Affiliations:** a West China College of Stomatology, Sichuan University, Chengdu 610041, Sichuan province, China; b Department of Biomedical Engineering, College of Engineering, Peking University, Beijing 100871, China; c Academy for Advanced Interdisciplinary Studies, Peking University, Beijing 100871, China; d Department of Applied Science and Technology, Saint Peter's College, New Jersey 07306, USA

**Keywords:** plasma, *Staphylococcus aureus*, *Enterococcus faecalis*, free radicals, reactive oxygen, ultraviolet

## Abstract

**Objective:**

A direct-current, cold atmospheric-pressure air plasma microjet (PMJ) was performed to inactivate *Staphylococcus aureus (S. aureus)* and *Enterococcus faecalis (E. faecalis)* in air. The process of sterilization and morphology of bacteria was observed. We wish to know the possible inactivation mechanisms of PMJ and explore a potential application in dental and other temperature sensitive treatment.

**Methods:**

In this study, we employed a direct current, atmospheric pressure, cold air PMJ to inactivate bacterias. Scanning electron microscopy was employed to evaluate the morphology of *S. aureus* and showed rupture of cell walls after the plasma treatment and Optical emission spectrum (OES) were used to understand the possible inactivation mechanisms of PMJ.

**Results:**

The inactivation rates could reach 100% in 5 min. When the distance between the exit nozzle of the PMJ device and Petri dish was extended from 1 cm to 3 cm, effective inactivation was also observed with a similar inactivation curve.

**Conclusion:**

The inactivation of bacteria is attributed to the abundant reactive oxygen and nitrogen species, as well as ultroviolet radiation in the plasma. Different life spans and defensibilities of these killing agents may hold the key to understanding the different inactivation curves at different treatment distances.

## INTRODUCTION

Atmospheric-pressure cold plasma has attracted considerable attention recently in the field of biomedical engineering, due to its extensive clinical applications, such as bacteria sterilization[1]–[3], cancer therapy[4],[5], blood coagulation[6],[7], wound healing[8],[9] and tooth whitening[10]. Non-thermal plasma, with gas temperature around or slightly above room temperature, generate many kinds of reactive species, such as excited atoms/molecules, charged particles and free radicals, such as hydroxyl radical (•OH), superoxide anion radical (•O_2_^−^), and singlet oxygen ([1]O_2_)[3],[11],[12], that can directly or indirectly interact with protein, lipid and nucleic acid molecules. It makes this system a good candidate for the sterilization of temperature sensitive materials and pathogenic bacteria which are resistant to traditional therapy.

In this study, we employed a direct current, atmospheric pressure, cold air plasma microjet (PMJ) to inactivate *Staphylococlus* (*S. aureus*) and *Enterococcus faecalis* (*E. faecalis*) in air. Different inactivation rates were observed when the distance between the PMJ and Petri dish was extended from 1 cm to 3 cm. Scanning electron microscope (SEM) and Optical emission spectrum (OES) were used to understand the possible inactivation mechanisms of PMJ.

## MATERIALS AND METHODS

### Plasma Device

**Fig. 1:**
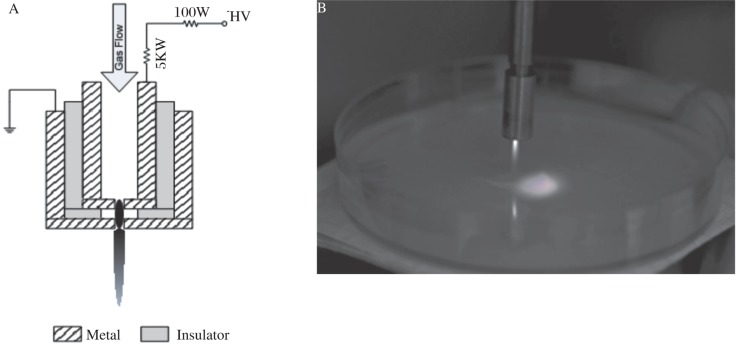
Plasma device. A: A schematic diagram of the device. B: A picture of PMJ sustained in air.

Two metal electrodes are separated from each other by a dielectric layer of ≤0.5 mm thickness. The openings in the two electrodes are ≤0.8 mm in diameter. The high-voltage electrode is completely embedded in the device and powered by a DC power supply (Matsusada AU5R120). The outer electrode is grounded for safety considerations. A schematic diagram of the device can be founded in ***Fig. 1A***. For detailed plasma device operation, please refer to references[2],[3]. We used compressed air as the working gas at a flow rate of approximately 5 slm. The discharge sustaining voltage was around 550 V with an operating current of 30 mA. Under these operating conditions, a PMJ of ≤1 cm visible length was generated. The gas temperature 1 cm away from the nozzle was measured to be around 38°C using a thermometer. Since the gas temperature decreased when the distance became longer, the temperature at 3 cm was not measured.

### Bacteria

*S. aureus* was purchased from the China General Microbiological Culture Collection Center. *E. faecalis* was a gift from Prof. Lihong Guo (Peking University). The bacteria were cultured in Luria-Bertani (LB) medium for 12-18 h until logarithmic growth phase. The suspensions were then diluted to a concentration of 10^4^ CFU (colony forming units) per mL, of which 150 µL suspension was spread uniformly on a LB agar culture medium in a Petri dish (90 mm in diameter) for plasma treatment and analysis.

After the treatment, plates were sealed and cultured in the incubator for 18-21 h at 37°C (*S. aureus*) or 30°C (*E. faecalis*). Subsequently, a CFU count was obtained on the Petri dish. The inactivation rate of the bacteria was defined as the percentage decrease in CFU counts of the plasma treated sample to that of the control.

### Plasma treatment

**Fig. 2:**
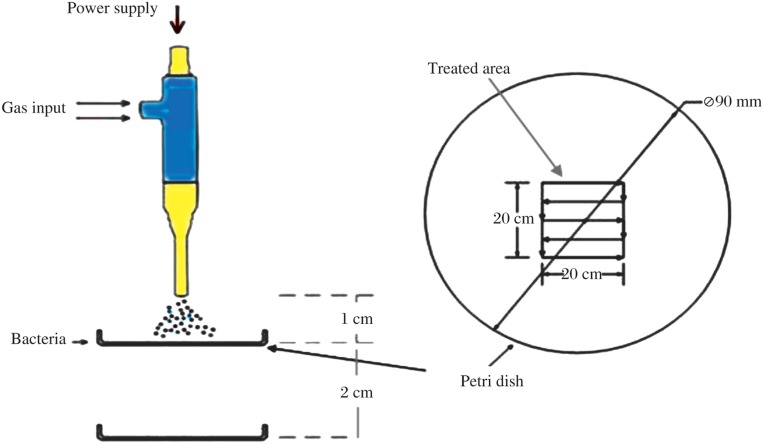
Schematic diagram of the PMJ treatment of a Petri dish[2]

A diagram of the treatment setup is shown in ***Fig. 2***. Treating distance was defined as the distance from the exit nozzle of the PMJ device to the surface of the Petri dish. Two distinct treating distances were used in this study, namely, 1 cm and 3 cm. The plasma treatment was limited to a 2 cm×2 cm square area in the center of the Petri dish (referred to as the “treated area”) (***Fig. 2***). For each bacteria sample, the Petri dish was moved under the plasma torch with a constant speed of 4 mm/s along the gridlines indicated in ***Fig. 2*** for a period of 30 s for *S. aureus* and 1 min for *E. faecalis*. The total treating time ranged from 30 s to 5 min. The experiment was repeated at least three times to obtain an average inactivation rate and a measure of variance. The control group was subjected only to air flow at the same flow rate. More detailed information can be found in our previous work[2].

### Optical emission spectrum

To identify the reactive species that are generated in the discharge and subsequently expelled, the OES in the visible region was recorded along the axial direction of the PMJ by a 0.75 m spectrometer (Princeton Instrument/Acton Spectra Pro 2750) together with an intensified CCD (Princeton Instrument I-MAX 512).

### Scanning electron microscopic imaging

Bacterial suspensions (20 mL) before and after treatment were centrifuged at 387.5 *g* for 20 min. The supernatant was discarded and the remaining film was treated by plasma (treated group) or air blown (control group). Both of the experimental group and control group were fixed overnight with 2.5% glutaraldehyde and then dehydrated sequentially in ethanol (30%,50%,70%,80%,90%,100%). The samples were gold-paladium coated and evaluated with a scanning electron microscope (Quanta 200FEG and NOVA NANOSEM 430). For the sake of better observation, the higher concentration of bacteria suspension was used, and the treating time was extended.

## RESULTS

### Inactivation rates

**Fig 3.**
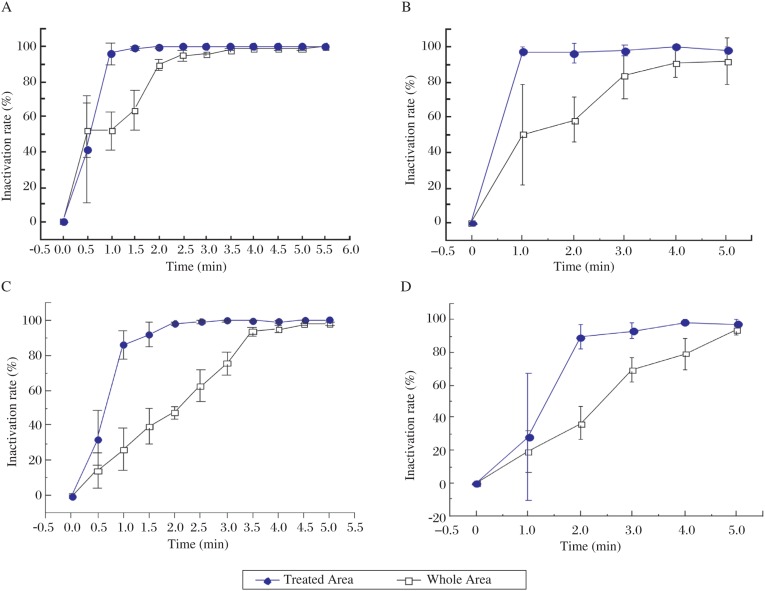
The inactination rate of two bacteria at different treating distance. The inactivation rate of *S. aureus* (A) and *E. faecalis* (B) at a treating distance of 1 cm. The inactivation rate of *S. aureus* (C) and *E. faecalis* (D) at a treating distance of 3 cm.

The inactivation rate of the bacteria was defined as the percentage decrease in CFU counts following treatment.***Fig. 3A and 3B*** shows the inactivation rates of *S. aureus* and *E. faecalis* at a treatment distance of 1 cm. Both inactivation rates reached 100% in 1 min in the treated area. Bacteria in other regions on the Petri dish were also inactivated. A 100% inactivation rate on the whole Petri dish was achieved in 4-5 min. This result was consistent with our previous work, where the inactivation of *S. aureus* and five other bacteria by PMJ were studied[2]. The slightly different inactivation effect of plasma on *S. aureus* and *E. faecalis* was speculated that there were differences in the resistance to inactivation of the different bacteria. When the treating distance was extended to 3 cm, similar inactivation trends were observed (***Fig. 3C and 3D***), although it took a longer time (2 min) to reach the 100% inactivation rate.

### Cell morphology evaluation

**Fig. 4:**
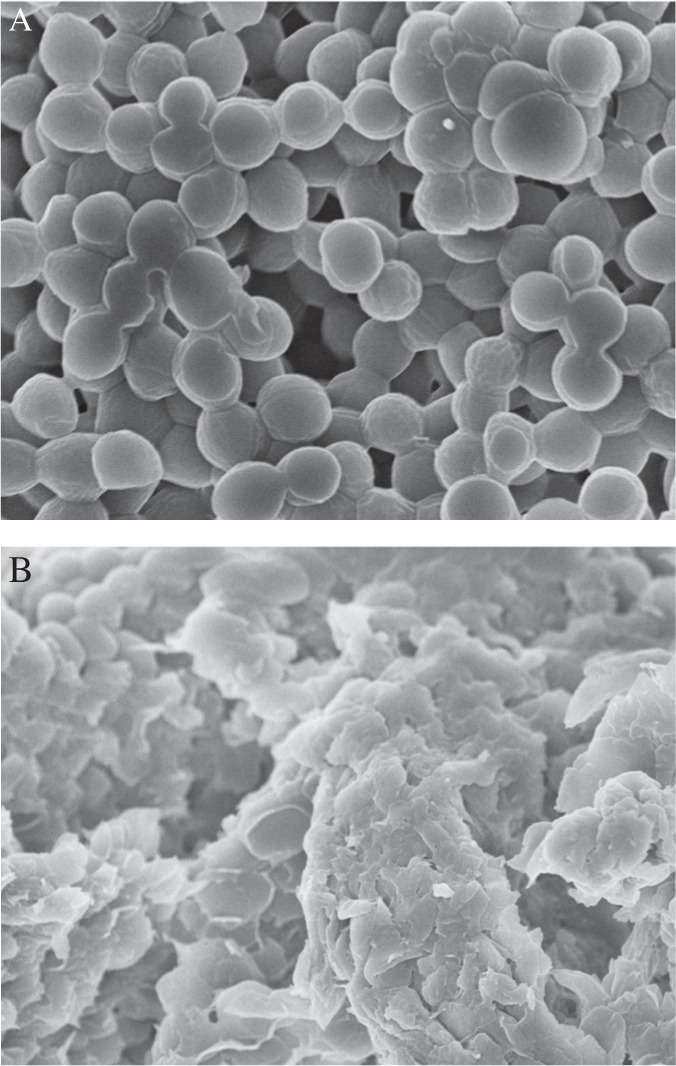
Scanning electron microscope photographs of *S. aureus* treated by PMJ. A: before treafed by PMJ. B: after treated by PMJ.

SEM was employed to evaluate the morphology of *S. aureus* before and after the PMJ treatment. ***Fig. 4B*** shows that the morphology of the bacteria underwent a transformation from their initial round, smooth surfaces to irregular structures with cytomembrane rupturing and even cytoplasm leakage after a 20 min PMJ treatment. The changes in the morphology of the cell wall were considered to be detrimental to the survival of the bacteria, accounting for the lethal effect on the bacteria of the PMJ.

### OES detection of air plasma

**Fig. 5:**
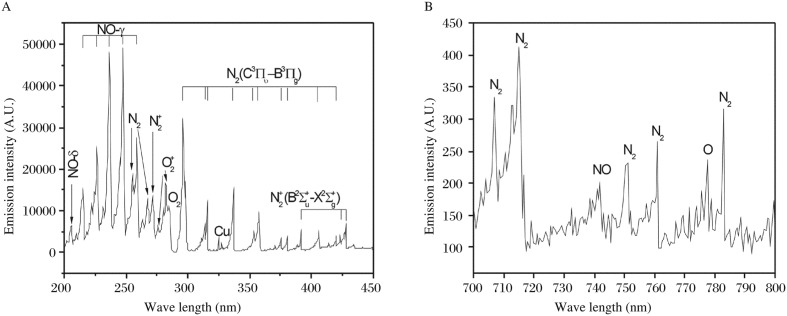
Emission spectrum of the PMJ at 200-450 nm (A) and 700-800 nm (B).

***Fig. 5A*** shows a typical emission spectrum of the PMJ taken along the direction from 200 nm to 450 nm, which is rich in spectroscopic information. We recognized the NO γ-system at 226.9 nm, 236.3 nm and 247.1 nm; N_2_ 2^+^ system at 337.1 nm, 357.7 nm and 380.5 nm; and N_2_^+^ 1^−^ system at 358.2 nm, 388.4 nm and 391.4 nm. We could not exclude some copper emission due to the use of copper as the electrode material. No clear OH emission was observed in the 306-309 nm region. ***Fig. 5B*** shows a near infrared spectrum where atomic oxygen emission at 777.2 nm and NO emission at 742.0 nm are easily identifiable.

## DISCUSSION

The atmospheric PMJ contains electrons, ions, excited atoms and molecules, reactive radicals, and radiation. The inactivation mechanisms for the death of microorganisms and the role of various inactivation agents are still under debate. Popular theories include erosion by free radicals, etching by charged particles and high energy electrons, and ultroviolet (UV) radiation damage[13].

Nitrogen oxide (NO) emissed in the UV region is the main source of agent for the inactivation of bacteria, which is also destructive to organisms, leading to dimerization of thymine bases in DNA strands, thus disturbing normal replication[14]. A few possible pathways for the generation of NO are listed below[15]:

N_2_ + O → NO + N

N + O_2_ → NO + O

N + O + N_2_ → NO + N_2_

NO + O_3_ ↔ NO_2_ + O_2_

NO_2_ + O_2_ + hv → O_3_ + NO

Atomic oxygen is very reactive and detrimental to biological molecules[16]. Protein molecules, as constituents of the cell membrane, are susceptible to oxidation by atomic oxygen or metastable oxygen molecules. Therefore, the drastic attack of atomic oxygen and singlet oxygen molecules may contribute to the erosion and finally to the rupture of the cell wall. Some other reactive species, such as •OH,•O_2_^−^,^1^O_2_, ozone (O_3_) and nitrogen dioxide (NO_2_), may also be produced in the PMJ. •OH can easily attack unsaturated fatty acids on the cell membrane[11]. •O_2_^−^ can mediate the generation of more reactive radicals, namely •OH and HOO•, and the latter can also initiate lipid peroxidation and DNA mutation[17]. ^1^O_2_ can oxidize unsaturated fatty acids and membrane proteins[12], while O_3_ can interfere with cellular respiration. All of these agents may contribute to the inactivation process.

As discussed by Mendis *et al*[18], charged particles such as ions or free electrons, should participate in the sterilization process. The layers of the cells may selectively absorb some charged particles of different electrical properties in different regions. The electric forces among them can exert pressure or stress, causing distortion, transmogrification and rupture of the cell walls. Furthermore, the cracks or holes induced by charged particles will facilitate the invasion of free radicals and UV radiation, thus accelerate the sterilization process.

*S. aureus* and *E. faecalis* follows different inactivation curves in the treated area and on the whole Petri dish, which may be attributed to the different distributions and diffusibilities of various germicidal agents, including charged particles, free radicals, UV photons and excited neutrals. The treated area received more UV radiation as well as more charged particle bombardment than in the periphery, which could partly account for the quick inactivation of bacteria in the treated area. However, some radicals have a longer lifetime and can thus travel laterally to the untreated area, leading to the gradual inactivation there. The inactivation of bacteria at 3 cm was slower than at 1 cm. A longer treating distance means higher recombination rates of charged particles as well as the decay of UV irradiation, leaving the main agent to be some long-lived radicals. The indirect and long-distance inactivation effect of plasma treatment implies its potential application in the disinfection of concealed slits, deep holes, and some other regions that are not readily accessible.

Nowadays, the eradication of persisting bacteria in the root canal system is the initial and fundamental goal of endodontic therapy, which is especially crucial for the final success of root canal therapy. Resistant microorganisms, such as gram-positive *E. faecalis*, are the main cause of failures. The conventional accepted treatment procedures to eliminate the infection include a combination of chemical cleaning involving irrigation with a disinfectant agent such as sodium hypochlorite (NaClO), and mechanical treatment with file that debride the root canal and produce a shaping effect[19]. But the latter is usually limited by the diverse and complex anatomy of the root canal system. NaClO can't completely sterilize bacteria in many cases[20]. Siqueira *et al*[21] tested the effect of 4% NaClO on *E. faecalis* after 5 min treatment, and concluded that 40% of the root canals still harbored viable bacteria after treatment. The limitations of chemo-mechanical therapy call for the development of new methods for the disinfection of the root canal, especially the lateral branch of the root canal and accessory root canal that are hardly reached by traditional methods because of the complex anatomy and depth. We expect this new technology can be applied clinically in root canal disinfection and thus bring forward a new era to oral health.

In conclusion, a direct-current, atmospheric-pressure cold air PMJ was successfully employed to inactivate *S. aureus* and *E. faecalis.* SEM images showed extreme cell morphology changes after plasma treatment, reflecting the lethal effect of plasma on bacteria. The inactivation rate increased with the exposure time, reaching 100% within 2 min treatment in the treated area. Although not directly exposed to PMJ, bacteria in the untreated area were also inactivated, with a longer time required to reach 100% inactivation. The effect of PMJ on bacteria at 1 cm was slightly better than that at 3 cm, which was speculated to be due to the stronger combined effect of charged particles, UV and free radicals. NO and atomic oxygen were detected in the emission spectrum, and were considered to be contributing to the sterilization. Other reactive species, such as •OH, •O_2_^−^, ^1^O_2_, and O_3_ were also believed to be involved in the inactivation process.
